# Supplementary material for: Light-oriented 3D printing of liquid crystal/photocurable resins and in-situ enhancement of mechanical performance
Source: Nat Commun. 2023 Oct 18;14:6586. doi: 10.1038/s41467-023-42369-1 (PMC10584836; doi:10.1038/s41467-023-42369-1)
Supplement: Supplementary file 1 — Supplementary Information [file 41467_2023_42369_MOESM1_ESM.pdf]

Supplementary information

Light-oriented 3D printing of liquid crystal/photocurable resins and in-situ enhancement of mechanical performance

Xiaolu Sun[1,2,3,4,†], Shaoyun Chen[1,2,3,4†*], Bo Qu[1,3,4], Rui Wang[1,3,4], Yanyu Zheng[1,3,4], Xiaoying Liu[1,3,4], Wenjie Li[1,3,4], Jianhong Gao[1,3,4], Qinhui Chen[2*]& Dongxian Zhuo[1,2,3,4*].

[1]College of Chemical Engineering and Materials Science, Quanzhou Normal University, Quanzhou, Fujian, 362000, P. R. China

[2]College of Chemistry and Materials Science, Fujian Normal University, Fuzhou, Fujian, 350007, P. R. China

[3]Fujian University Engineering Research Center of Polymer Functional Coating based Graphene, Fujian, 362000, P. R. China

[4]Fujian Key Laboratory of New Materials for Light Textile and Chemical Industry, Fujian, 362000, P. R. China

† These authors contributed equally: Xiaolu Sun, Shaoyun Chen

*Corresponding Authors: chshaoy@qztc.edu.cn; chenqh@fjnu.edu.cn; dxzhuo@qztc.edu.cn;

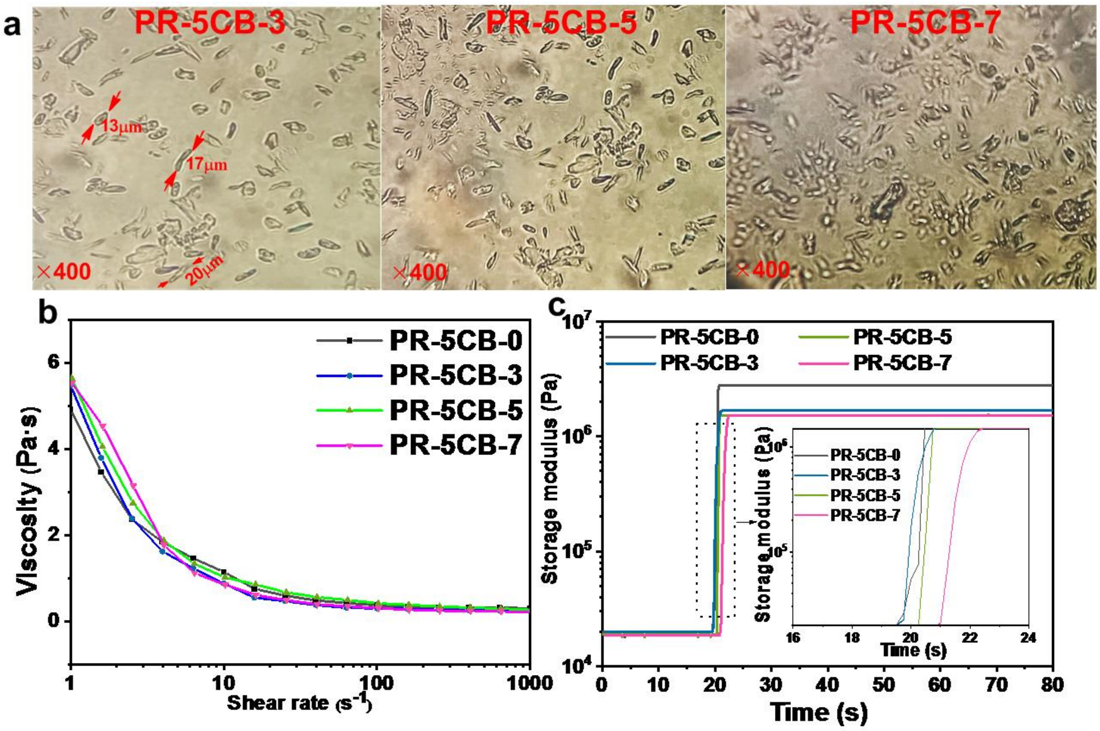

Supplementary Fig. 1: Dispersibility and rheological properties of PR-5CB resins. a Polarizing optical microscope (POM) diagrams of PR-5CB resins at 30 ℃. **b** Relationship between viscosity and shear rate for PB-5CB resins. **c** Kinetics of the curing reaction for the PR-5CB resins.

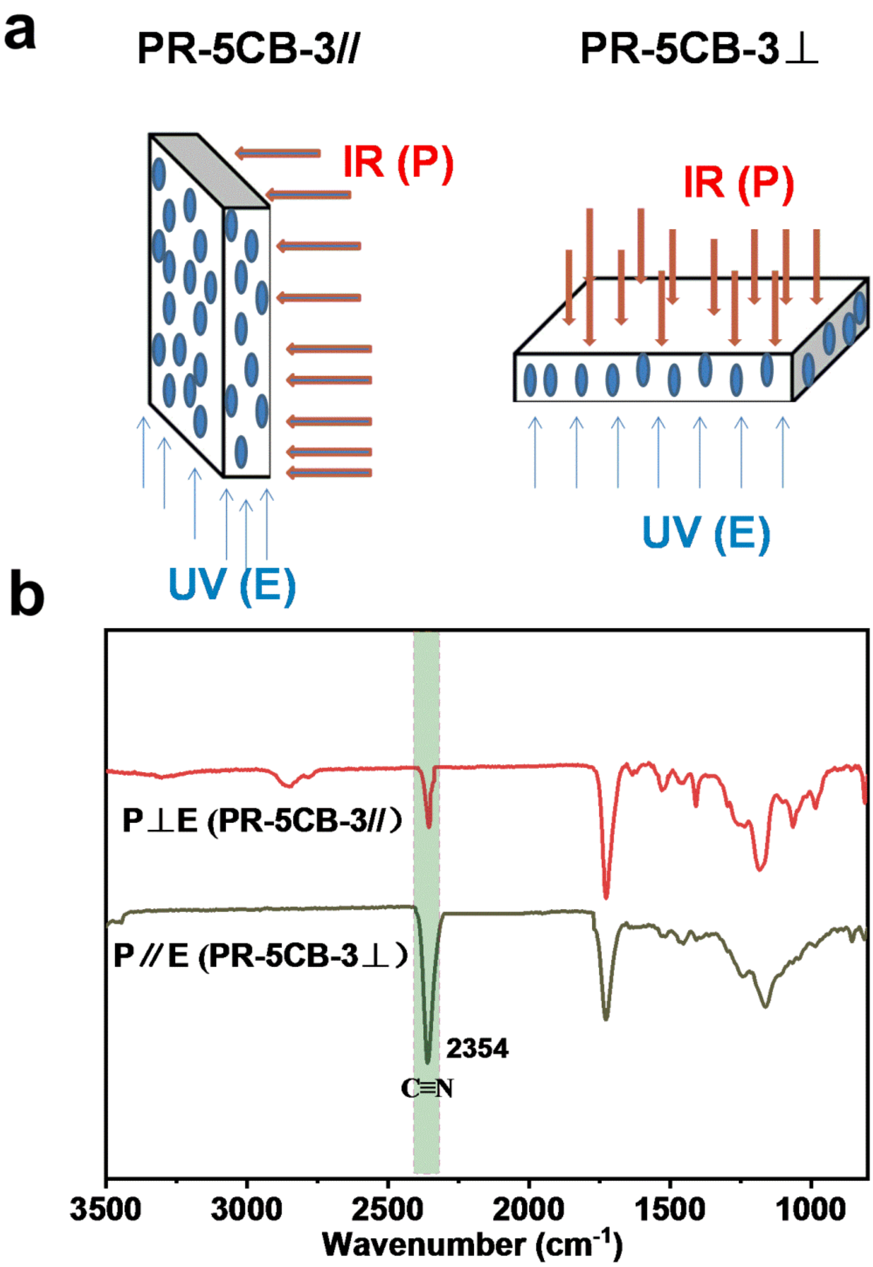

Supplementary Fig.2: a Different methods of printing IR test films. **b** FTIR spectra of the films. (P is the polarization direction of the IR polarized light and E is the vibration vector of the polarized UV light.)

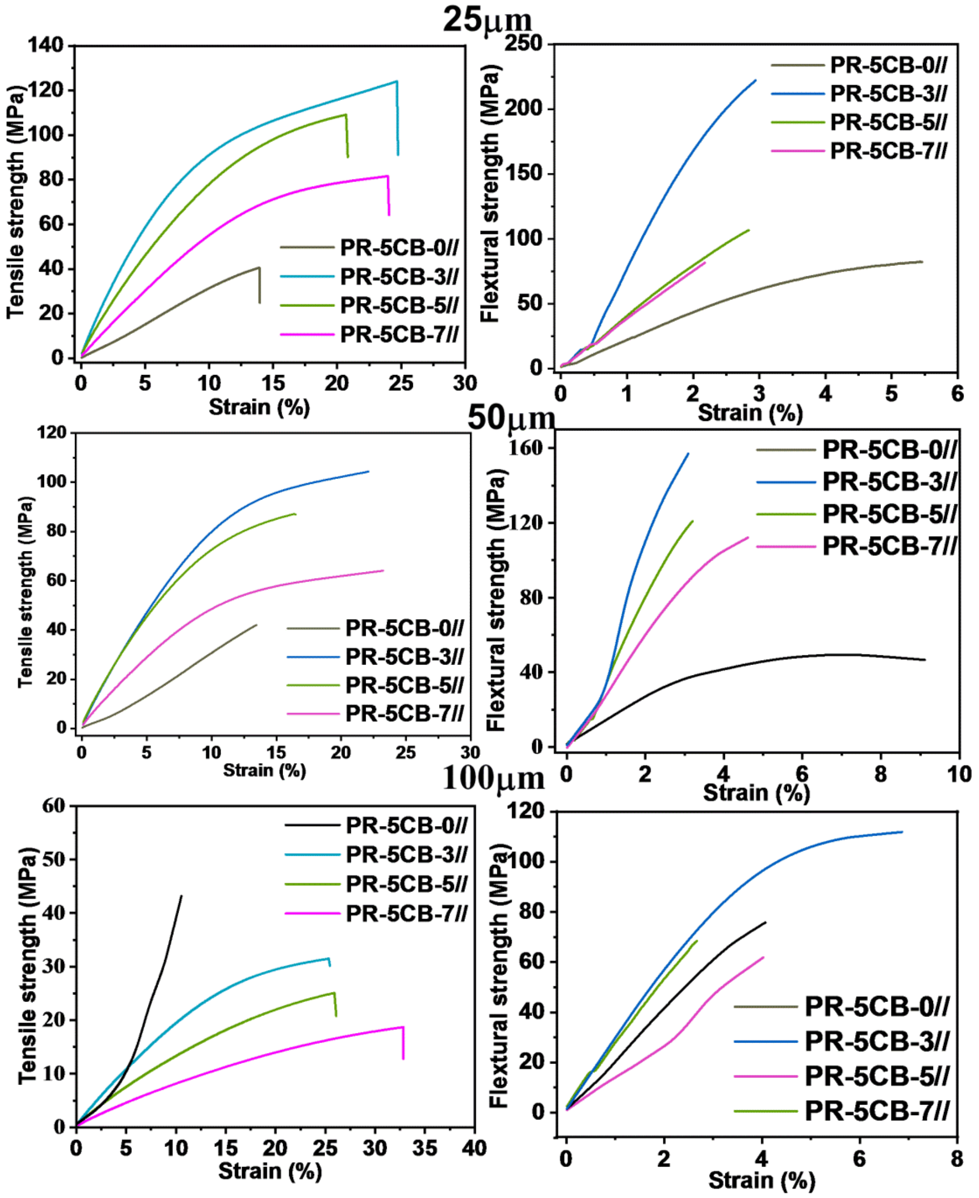

Supplementary Fig. 3: Stress- strain curves and bending- strain curves of 3D printed samples obtained using PR-5CB resins with different printing resolutions.

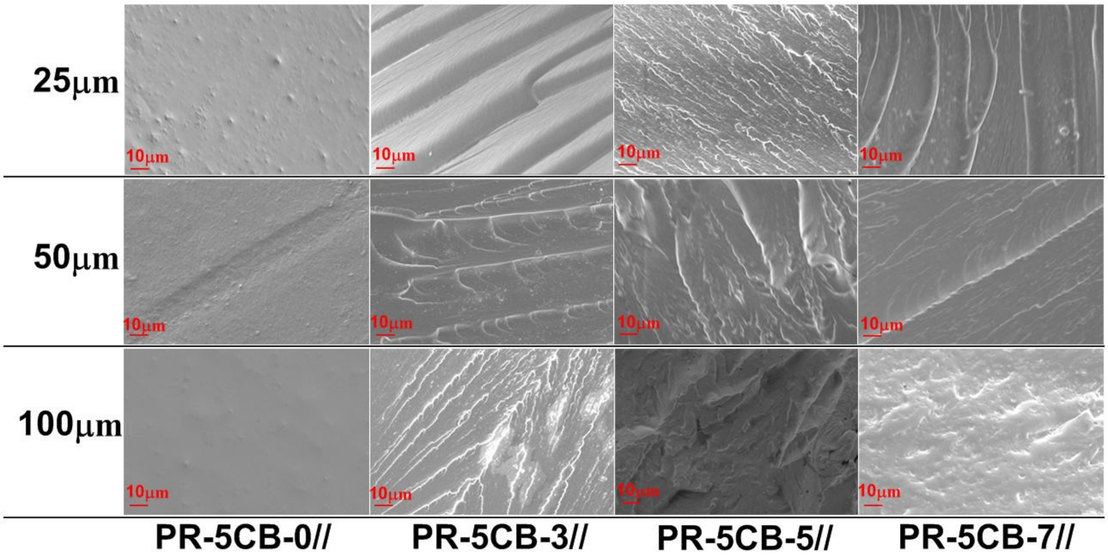

Supplementary Fig. 4: SEM images of fracture surface morphology of the 3D-printed parts based PR-5CB resins with different print resolutions.

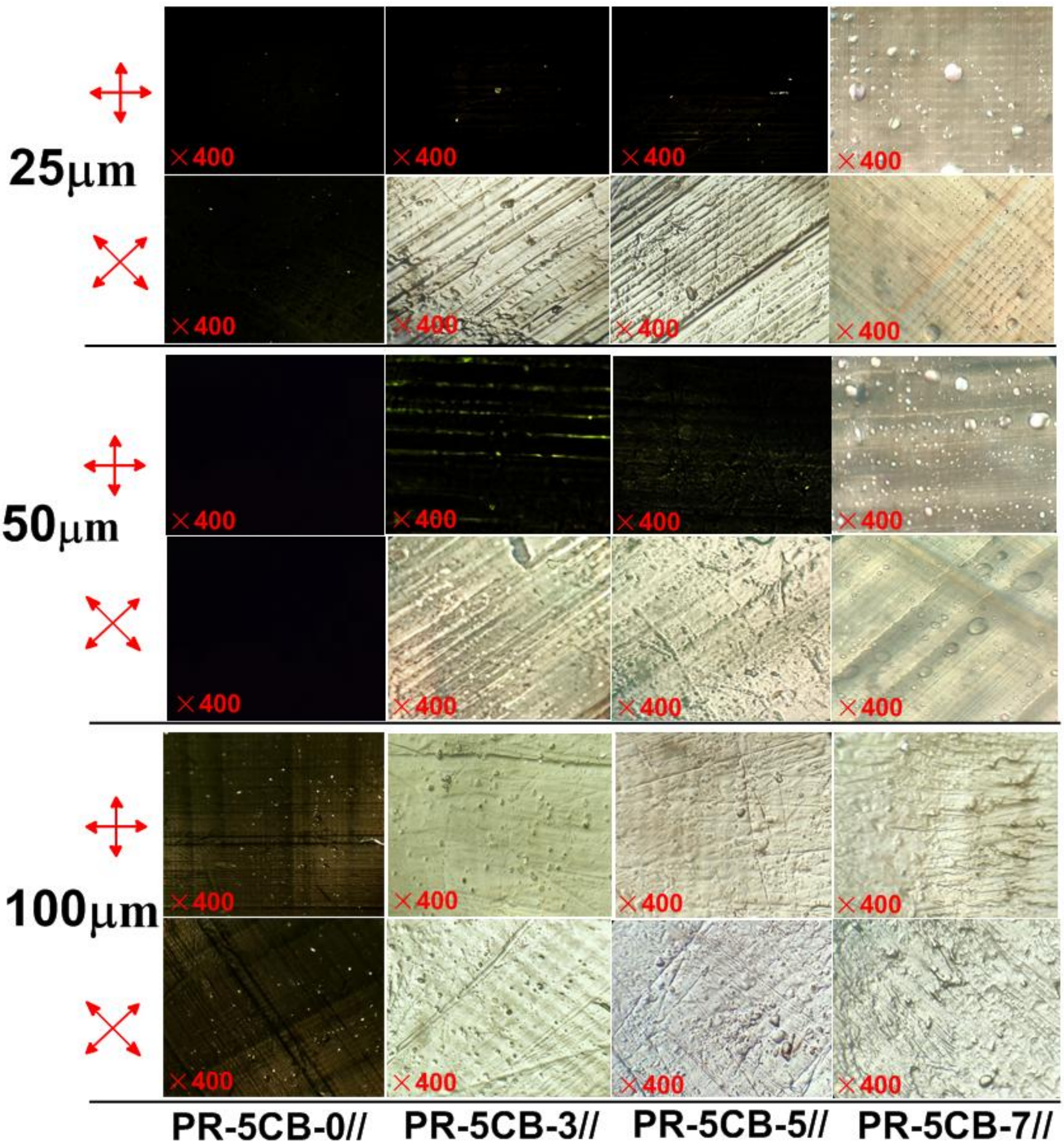

Supplementary Fig. 5: POM images of the 3D-printed parts surface based PR-5CB resins at different print resolutions.

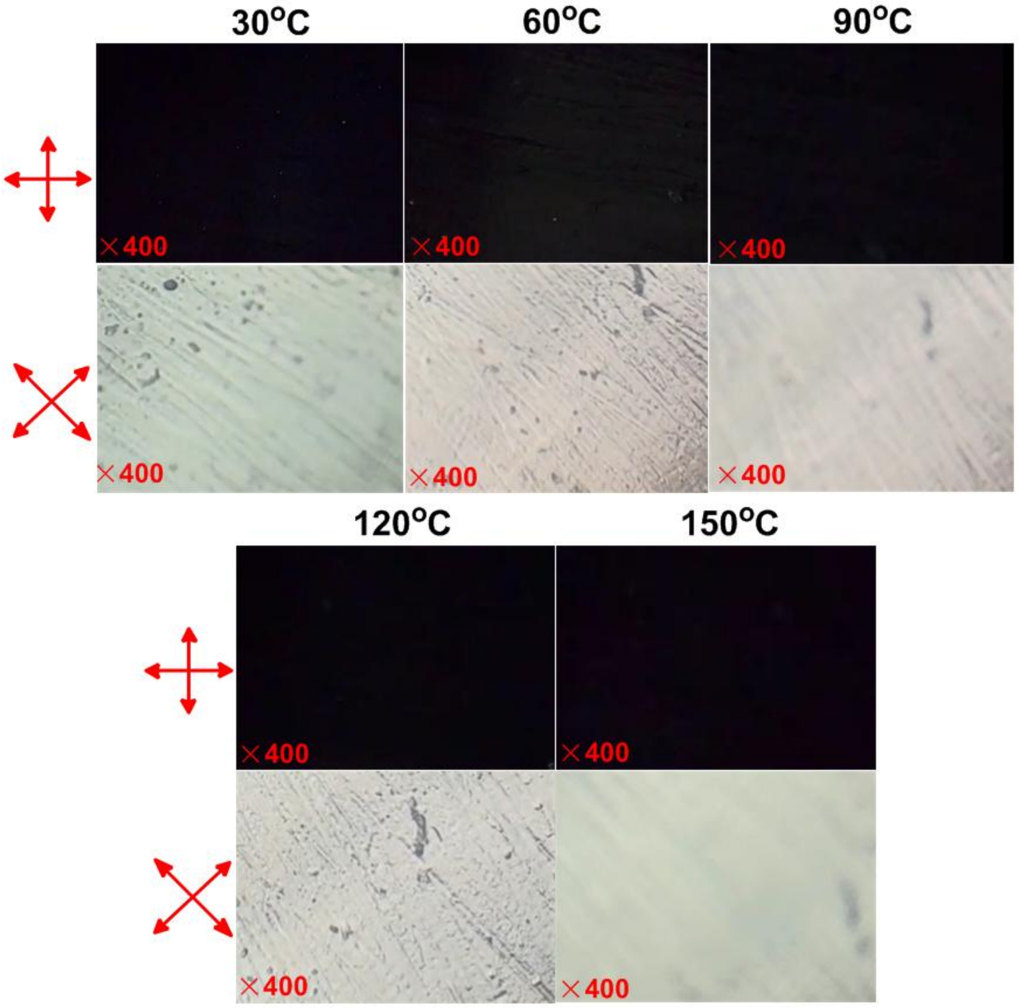

Supplementary Fig.6: POM images of PR-5CB-3// (25 μm) resin surface at different temperatures.

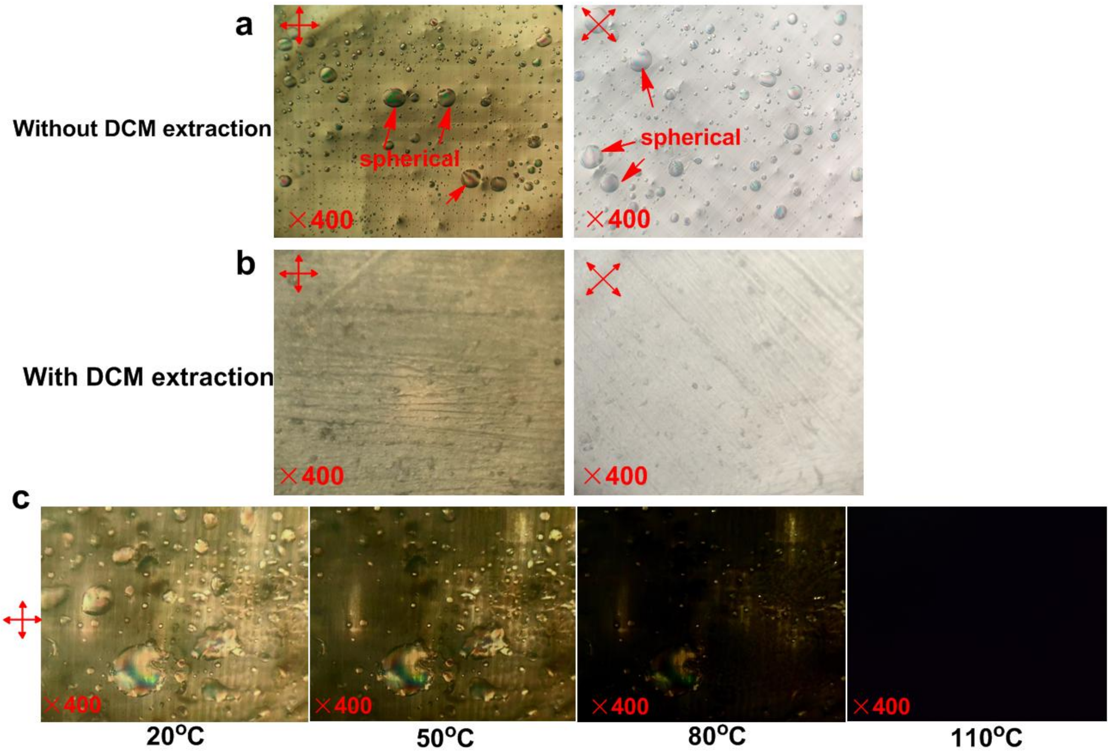

Supplementary Fig.7: Verifying the spherical LCs immobilizing on the surface of the samples. **a** POM of PR-5CB-7// (50 μm) without extracting 5CB. **b** POM of PR-5CB-7// (50 μm) with extracting 5CB. **c** POM images of PR-5CB-7// (50 μm) resin surface at different temperatures.(Dichloromethane (DCM))

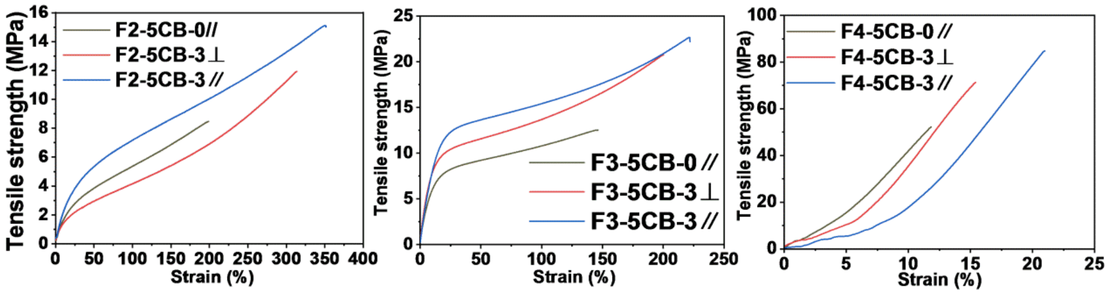

Supplementary Fig.8: Stress-strain curves of different commercial printing formulations with 3% content of 5CB

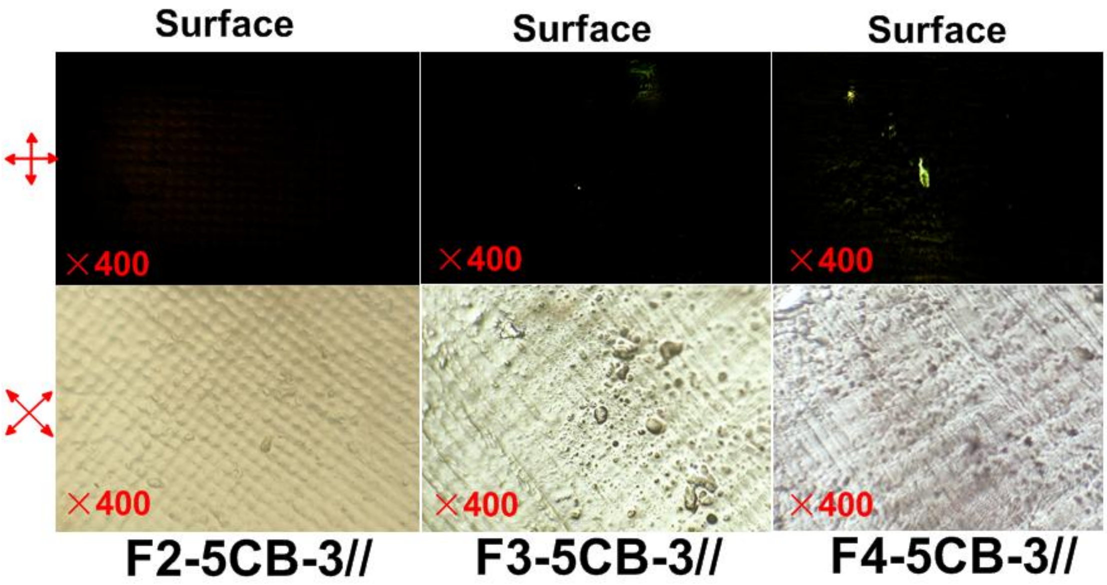

Supplementary Fig.9: Polarizing optical microscopy (POM) at 0° and 45° of different commercial printing formulations with 3% content of 5CB

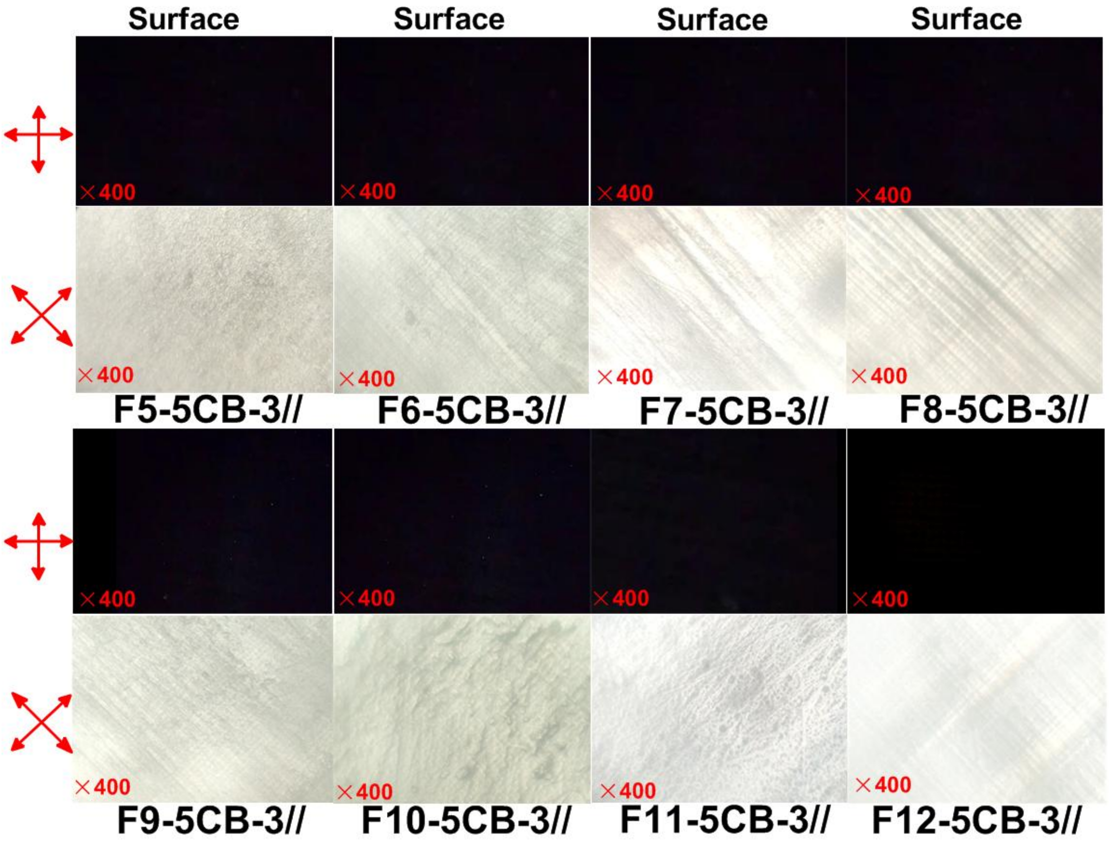

Supplementary Fig.10: Polarizing optical microscopy (POM) at 0° and 45° of different prepolymer printing formulations with 3% content of 5CB

Supplementary Table 1: Formulations of PR-5CB resins.

Materials	PR-5CB -0	PR-5CB–3	PR-5CB -5	PR-5CB -7
5CB	0	3.0	5.0	7.0
CN9010	30.0	30.0	30.0	30.0
CN991	30.0	30.0	30.0	30.0
SR494	15.0	15.0	15.0	15.0
HEMA	20.0	20.0	20.0	20.0
TPO	5.0	5.0	5.0	5.0

Supplementary Table 2: Comparison of mechanical and thermal properties of 3D-printed samples based on different photosensitive resins.

**Samples**	**Tensile Strength (MPa)**	**Elongation at Break (%)**	**Reference**
SiO_2 filling	53.8	2.7	[1]
Calcium sulfate whiskers filling	29.0	4.8	[2]
Graphene oxide filling	61.9	7.2	[3]
polysiloxane core-shell nanoparticles	53.6	12.84	[4]
Multi-walled carbon	57.0	9.9	[5]
Polyimide	24.9	5.4	[6]
ALCR-2	65.3	17.3	[7]
PR-5CB-3//	122.2	23.4%	This work

Supplementary Table 3: Different commercial printing formulations with 3% content of 5CB

F2	Aliphatic polyether acrylates	CN991	HPMA	TPO	
	46	5	46	3	
F3	Aliphatic polyether acrylates	CN991	HEMA	HPMA	TPO
	36	5	20	36	3
F4	CN9010	CN991	HEMA	HPMA	TPO
	40	32	15	10	3

Supplementary Table 4: Tensile results of the 3D printing strips based on F2, F3 and F4

		Tensile Strength/MPa	Elongation at break/%
F2	F2-5CB-0//	8.5	198.8
	F2-5CB-3⊥	11.9	313.6
	F2-5CB-3//	15.0	351.6
F3	F3-5CB-0//	12.5	146.2
	F3-5CB-3⊥	20.7	200.1
	F3-5CB-3//	22.2	221.98
F4	F4-5CB-0//	11.8	52.2
	F4-5CB-3⊥	15.4	71.3
	F4-5CB-3//	21.0	84.8

Supplementary Table 5: Formulation of various prepolymers with 3% 5CB photosensitive resins

F5	CN991	HPMA	TPO	
	62	35	3	
F6	Aliphatic polyether acrylates	HEMA	TPO	
	67	30	3	
F7	CN9010	HEMA	TPO	
	58	39	3	
F8	Aliphatic polyether acrylates	HEMA	HPMA	TPO
	50	20	27	3
F9	Aliphatic polyether acrylates	CN991	HEMA	TPO
	30	35	32	3
F10	CN9010	HEMA	HPMA	TPO
	57	10	30	3
F11	CN9010	CN991	HEMA	TPO
	32	28	37	3
F12	SR494	HEMA	HPMA	TPO
	50	30	17	3

Supplementary Methods

Fourier transform polarization infrared spectroscopy

A Nicolet-iS10 infrared spectrometer (USA) was utilized to probe the arrangement pattern of liquid crystal molecules. The Attenuated Total Reflection (ATR) mode was used, the scanning range was 4000~400 cm-1, the resolution was 0.2 cm^{-1}, and the number of scans was 32. PR-5CB-3 was selected to print films with a thickness of 150 μm in different directions, and the polarized infrared absorption spectra were observed on the surface of the printed films printed in different directions.

Supplementary References

1. Weng, Z., Zhou, Y., Lin, W., Senthil, T. & Wu, L. Structure-property relationship of nano enhanced stereolithography resin for desktop SLA 3D printer. *Compos. Part A. Appl. Sci. Manuf.* **88**, 234–242 (2016).

2. Liu, Y., Lin, Y., Jiao, T., Lu, G. & Liu, J. Photocurable modification of inorganic fillers and their application in photopolymers for 3D printing. *Polym. Chem. UK.* ***10***, 6350–6359 (2019).

3. Ramírez-Soria, E. et al. On the effect of ultralow loading of microwave-assisted bifunctionalized graphene oxide in stereolithographic 3D-printed nanocomposites. *ACS Appl. Mater. Inter.* **12**, 49061–49072 (2020).

4. Li, Y. et al. Structure-property relationship of stereolithography resins containing polysiloxane core-shell nanoparticles. *ACS Appl. Mater. Inter.* **12**, 4917–4926 (2020).

5. Hector Sandoval, J. & Wicker, R. B. Functionalizing stereolithography resins: effects of dispersed multi-walled carbon nanotubes on physical properties. *Rapid. Prototyp. J.* **12 (5)**, 292–303 (2006).

6. Guo, Y., Ji, Z., Zhang, Y., Wang, X. & Zhou, F. Solvent-free and photocurable polyimide inks for 3D printing. *J Mater. Chem. A.* **5**, 16307–16314 (2017).

7. Chen, S. et al. Synthesis of novel acrylic liquid-crystal resin and its in-situ enhancement in light-curing 3D printing performance. *J. Mater. Res. Technol.* **17**, 2158–2174 (2022).
