# Supplementary material for: Light-oriented 3D printing of liquid crystal/photocurable resins and in-situ enhancement of mechanical performance
Source: Nat Commun. 2023 Oct 18;14:6586. doi: 10.1038/s41467-023-42369-1 (PMC10584836; doi:10.1038/s41467-023-42369-1)
Supplement: Supplementary file 3 — Description of Additional Supplementary Files [file 41467_2023_42369_MOESM3_ESM.pdf]

Description of Additional Supplementary Files

Supplementary Movie 1: POM of the 3D-printed products with PR-5CB-0//(25 µm).

Supplementary Movie 2: POM of the 3D-printed products with PR-5CB-0⊥(25 µm).

Supplementary Movie 3: POM of the surface of 3D-printed products with PR-5CB-3//(25 µm).

Supplementary Movie 4: POM of the cross section of 3D-printed products with PR-5CB-3//(25 µm).

Supplementary Movie 5: POM of the surface of 3D-printed products with PR-5CB-3⊥(25 µm).

Supplementary Movie 6: POM of the cross section of 3D-printed products with PR-5CB-3⊥(25 µm).

Supplementary Movie 7: POM of the 3D-printed products with PR-5CB-3//(25 µm) after extracting 5CB.

Supplementary Movie 8: POM of the 3D-printed products with F2-5CB-3//(25 µm).

Supplementary Movie 9: POM of the 3D-printed products with F3-5CB-3//(25 µm).

Supplementary Movie 10: POM of the 3D-printed products with F4-5CB-3//(25 µm).
